# THE ROLE OF COMMUNITY-BASED ORGANIZATIONS IN SUPPORTING HEALTHY AGING

**DOI:** 10.1093/geroni/igae098.1186

**Published:** 2024-12-31

**Authors:** Rebecca White, Andrew Sixsmith, mei Fang

**Affiliations:** Simon Fraser University, Vancouver, British Columbia, Canada; Simon Fraser University, Vancouver, British Columbia, Canada; SIMON FRASER UNIVERSITY, Simon Fraser University, British Columbia, Canada

## Abstract

Maintaining a healthy and balanced lifestyle is crucial for healthy aging and has a significant positive impact on brain health. Community-based organizations play an important role in many countries in promoting healthy lifestyles, particularly among those with the greatest social and economic need, through the provision of social, recreational, and informational programming. This presentation draws on research carried out in Vancouver, Canada, to understand the role of community-based organizations in enhancing the physical, social, and emotional well-being of older people. Using an institutional ethnographic case study approach guided by principles of community-based participatory research, the research focuses on the role of community-based organizations in enhancing older people’s well-being at multiple levels and contextualizes such organizations within a broader healthcare ecosystem. Results demonstrate the multi-faceted role community-based organizations play in supporting health and well-being among older people. The shift towards integrated healthcare models with a more holistic approach to health presents opportunities for community-based organizations to assume a more prominent role in empowering older people to self-manage their health and promote healthy lifestyles. Yet, such organizations face a multitude of challenges, particularly securing sustainable operational funding and widening their appeal to a changing demographic. The research highlights how community assets may be mobilized to better support the health and well-being of aging communities. More research is needed to understand how community-based organizations can better communicate their value and impact and enhance their capacity to respond effectively to emerging opportunities.

